# Dose-Dependent Pattern of Cochlear Synaptic Degeneration in C57BL/6J Mice Induced by Repeated Noise Exposure

**DOI:** 10.1155/2021/9919977

**Published:** 2021-06-09

**Authors:** Minfei Qian, Qixuan Wang, Zhongying Wang, Qingping Ma, Xueling Wang, Kun Han, Hao Wu, Zhiwu Huang

**Affiliations:** ^1^Department of Otolaryngology-Head and Neck Surgery, Ninth People's Hospital, Shanghai Jiao Tong University School of Medicine, Shanghai 200011, China; ^2^Ear Institute, Shanghai Jiao Tong University School of Medicine, Shanghai 200011, China; ^3^Shanghai Key Laboratory of Translational Medicine on Ear and Nose Diseases, Shanghai 200011, China; ^4^Department of Otolaryngology, Ren-Ji Hospital, Shanghai Jiao Tong University School of Medicine, Shanghai 200127, China

## Abstract

It is widely accepted that even a single acute noise exposure at moderate intensity that induces temporary threshold shift (TTS) can result in permanent loss of ribbon synapses between inner hair cells and afferents. However, effects of repeated or chronic noise exposures on the cochlear synapses especially medial olivocochlear (MOC) efferent synapses remain elusive. Based on a weeklong repeated exposure model of bandwidth noise over 2-20 kHz for 2 hours at seven intensities (88 to 106 dB SPL with 3 dB increment per gradient) on C57BL/6J mice, we attempted to explore the dose-response mechanism of prolonged noise-induced audiological dysfunction and cochlear synaptic degeneration. In our results, mice repeatedly exposed to relatively low-intensity noise (88, 91, and 94 dB SPL) showed few changes on auditory brainstem response (ABR), ribbon synapses, or MOC efferent synapses. Notably, repeated moderate-intensity noise exposures (97 and 100 dB SPL) not only caused hearing threshold shifts and the inner hair cell ribbon synaptopathy but also impaired MOC efferent synapses, which might contribute to complex patterns of damages on cochlear function and morphology. However, repeated high-intensity (103 and 106 dB SPL) noise exposures induced PTSs mainly accompanied by damages on cochlear amplifier function of outer hair cells and the inner hair cell ribbon synaptopathy, rather than the MOC efferent synaptic degeneration. Moreover, we observed a frequency-dependent vulnerability of the repeated acoustic trauma-induced cochlear synaptic degeneration. This study provides a sight into the hypothesis that noise-induced cochlear synaptic degeneration involves both afferent (ribbon synapses) and efferent (MOC terminals) pathology. The pattern of dose-dependent pathological changes induced by repeated noise exposure at various intensities provides a possible explanation for the complicated cochlear synaptic degeneration in humans. The underlying mechanisms remain to be studied in the future.

## 1. Introduction

Noise-induced hearing loss (NIHL) is a global public health issue. Hearing loss could be caused by genetic factors, aging, infectious diseases, ototoxic drugs, and noise exposure [1–6]. The reported mechanisms of noise-induced hair cells (HCs) and spiral ganglion neuron damage mainly include mechanical shearing forces and oxidative damage to HCs [7] and glutamate excitotoxicity to neurons [8–11]. In the past, noise exposure was considered harmful only when it causes a permanent threshold shift (PTS) [3, 12–16]. However, Kujawa and Liberman recently demonstrated that even a single acute noise exposure at moderate intensity that induces temporary threshold shift (TTS) could result in permanent loss of ribbon synapses, which was then known as synaptopathy [17]. Noise-induced cochlear synaptopathy has been the focus of attention in hearing research in these years. A number of studies further found that the loss of ribbon synapse between cochlear inner hair cells and type I afferent nerve (AN) fibers usually accompanies the abnormal suprathreshold auditory brainstem response (ABR) [17–19]. More and more evidences indicate that cochlear synaptopathy might not only be the primary mechanism of hidden hearing loss (HHL) but also contribute to tinnitus and age-related hearing loss (ARHL) [20–23].

Despite the large number of pathological studies involving the effects of noise on the cochlear synaptopathy, most of the previous studies paid attention to the single or acute noise exposure that induced TTS with the loss of ribbon synapses [17, 19, 24, 25]. Although repeated noise exposure is more common in human daily life (such as noise at bars, cinemas, concerts, and traffics), relatively few studies focused on the effects of repeated or chronic noise exposures on the cochlear synaptopathy [26–28]. It remains inconsistent whether repeated noise exposure would cause more damage on cochlear synapses, since the noise exposure procedure and experimental animals in previous studies were quite various. For instance, 16-week-old Sprague-Dawley rats exposed to 8-16 kHz octave-band noise at 97 dB SPL for 2 hours in 4 repeated days did not produce similar ABR wave I amplitude decrement as acute noise exposure [27], while repeated white noise at 100 dB SPL for 2 hours even cause additional cochlear damages in C57BL/6J mice [26]. Moreover, a recent study suggested that the medial olivocochlear (MOC) efferent feedback protects the cochlea from loss of ribbon synapses under the weeklong exposure to moderate-intensity noise (84 dB SPL) in mice [29], while few studies reported chronic noise exposure-induced MOC efferent synaptic degeneration.

In this study, to explore the pattern of cochlear afferent and efferent synaptic degeneration induced by repeated noise exposure, we used seven gradient levels of noise exposure at low, moderate, and high intensity. Auditory function and cochlear immunofluorescence were measured at baseline and 1 day and 14 days post noise exposure to assess the pathological changes of ribbon and MOC efferent synapses in C57BL/6J mice. We proposed a hypothesis that dose-dependent cochlear synaptic degeneration in C57BL/6J mice was induced by repeated noise exposure.

## 2. Materials and Methods

### 2.1. Animals

C57BL/6J mice aged four weeks were obtained from the SIPPR-BK Laboratory Animal, Ltd. (Shanghai, China), which were derived from breeders originally purchased from The Jackson Laboratory. A total of about 82 male mice were used in this study to exclude potential sex differences in susceptibility to NIHL [30]. Animals were divided into one control group and seven experimental groups. Mice were housed under quiet laboratory conditions, which showed normal baseline ABR, and distortion product otoacoustic emission (DPOAE) thresholds were included in subsequent noise exposure experiments. Each group included 8-12 mice in the analyses. All experimental procedures followed the *Guide for the Care and Use of Laboratory Animals* and were approved by the University Ethics Committee for Laboratory Animals of Shanghai Jiao Tong University.

### 2.2. Repeated Noise Exposure Procedure

**Figure 1:**
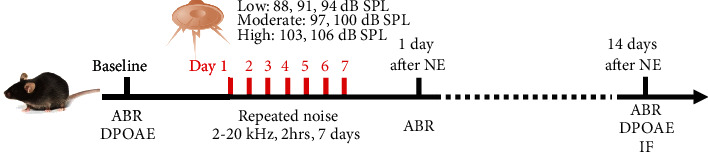
Flowchart of the repeated noise exposure procedure. NE: noise exposure; ABR: auditory brainstem response; DPOAE: distortion product otoacoustic emission; IF: immunofluorescence.

Noise exposure was performed by exposing conscious mice in a pie-shaped wire cage separated by eight compartments, in a calibrated reverberating chamber as described previously [31]. Bandpass-filtered noise of 2-20 kHz generated by MATLAB software (version 2007b) was delivered for 2 hours by an amplifier and loudspeaker (Yamaha) at seven gradients of intensity from 88 to 106 dB SPL. We defined the groups of relatively low intensity (88, 91, and 94 dB SPL), moderate intensity (97 and 100 dB SPL), and high intensity (103 and 106 dB SPL) in this study. An acoustimeter (type AWA6228+, Hangzhou Aihua) was used to calibrate noise exposure to the target sound pressure level. The exposure procedure was performed on seven exposed groups and their corresponding control groups of mice for continuous seven days repeatedly. The baseline was set at the day before the first day of noise exposure, ABRs were performed at baseline and 1 day and 14 days after NE, and DPOAEs were performed at baseline and 14 days after NE. Animals were sacrificed 14 days after NE (aged seven weeks) for observation of cochlear morphology using immunofluorescence (IF).  Figure 1 shows the flowchart of the repeated noise exposure procedure.

### 2.3. ABR Tests

ABRs were performed at baseline and 1 day and 14 days after the repeated noise exposure procedure. Mice were anesthetized with xylazine (20 mg/kg) and ketamine (100 mg/kg) through intraperitoneal injection, and the body temperature was maintained near 37°C using a heating blanket (Harvard Apparatus, USA, 55-7020). Recordings were performed using three subcutaneous needle electrodes at the vertex (active), left mastoid area (reference), and right shoulder (ground), respectively. Short tone burst stimuli of 3 ms duration with 1 ms rise/fall times were generated by the RZ6 workstation (Tucker-Davis Technologies, USA). Stimulus sounds were delivered free-field via an MF-1 speaker placed 10 cm away from the vertex, in front of the mouse. Stimulus roved over frequencies of 32, 22.6, 16, 11.3, 8, and 4 kHz, and the sound level started from 90 to 0 dB sound pressure level (SPL) in 5 dB steps. For each ABR waveform, 400 responses were collected and averaged. ABR thresholds were identified as the minimal stimulus level that evoked any noticeable recording of waveforms at each frequency. Wave I amplitudes (*μ*V) were measured by averaging the Δ*V* of both sides of the peak using the BioSigRZ software (Tucker-Davis Technologies, USA). Thresholds and amplitudes were measured by a researcher who was blind to the information of mice in groups.

### 2.4. DPOAE Tests

DPOAEs were performed at baseline and 14 days after repeated noise exposure of groups 88 dB SPL (representative low intensity) and moderate and high intensities, by the DPOAE workstation with BioSigRZ software (Tucker-Davis Technologies). For recordings, the left external auditory meatus of mice was coupled to a ER10B+ microphone (Etymotic Research). Two MF-1 speakers were used to deliver equal intensity primary tones, and the frequency ratio (*f*_2_/*f*_1_) was 1.2 of which [32]. The amplitude of distortion product (DP) at the frequency 2*f*_1_‐*f*_2_ was collected and averaged 512 times, in response to centre frequencies at 8, 16, and 22.6 kHz presented from 80 to 20 dB SPL (in 5 dB increments). The DPOAE threshold was defined as the point where the DP can no longer be detected from noise [33]. Thresholds were measured by a researcher who was blind to the information of mice in groups.

### 2.5. Whole-Mount Cochlear Immunofluorescence

At 14 days after the noise exposure procedure, mice in different noise-exposed groups and nonexposed control groups were deeply anesthetized and sacrificed. Cochleae were immediately dissected from temporal bones in 10 mM phosphate buffer saline (PBS) solution (Sigma, USA) and then perfused with 4% paraformaldehyde (Sigma, USA) at 4°C overnight. The fixed cochlea was decalcified in 10% ethylene diamine tetraacetic acid (Sigma, USA) solution until it became boneless. The organ of Corti was dissected from the decalcified cochlea and then separated into three parts (the apical, middle, and basal turn) in the PBS solution. For immunofluorescence (IF), the tissue was blocked in 10% bovine serum albumin solution with 0.3% Triton X-100 (Sigma, USA) for one hour at room temperature. Primary antibodies mainly included rabbit anti-myosin VIIa (Abcam, UK, 1 : 500), mouse anti-CtBP2 immunoglobulin (Ig) G1 (BD Biosciences, USA, 1 : 200), and rabbit anti-synaptophysin (SYP) (Abcam, UK, 1 : 500). Secondary antibodies used were Alexa Fluor 633-conjugated goat anti-mouse IgG1 and Alexa Fluor 488-conjugated goat anti-rabbit IgG (Invitrogen, USA, 1 : 200). Images were acquired using a 40x water or 63x oil objective lens on a LSM880 confocal microscope (Carl Zeiss, Germany) with Z-stack scanning for 10 *μ*m. The maximum intensity projection analysis was performed by using Zen software (Carl Zeiss, Germany, version 3.0).

### 2.6. Morphometric Analysis

**Figure 2:**
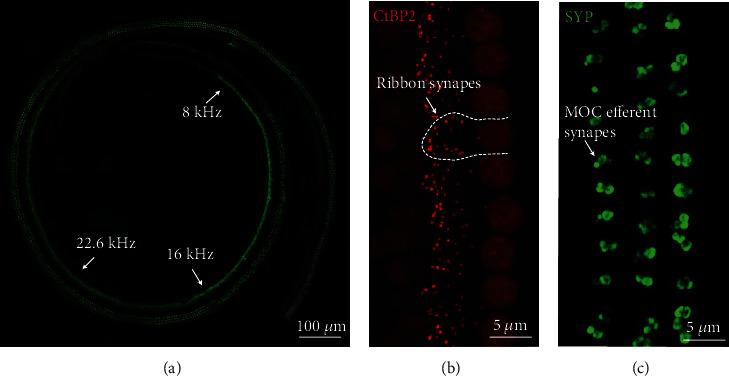
Morphometric analysis of frequency located ribbon and MOC efferent synapses: (a) frequency mapping on the organ of Corti; (b) ribbon synaptic counting; (c) MOC efferent synaptic measurement.

In order to map the location of specific frequency on the organ of Corti in mice, we used a location-to-frequency relationship formula as previous studies described [34, 35]: *d* (%) = 156.5‐82.5 × log(*f*), where *d* is the percentage of the distance from the base and *f* is the frequency in kHz. For morphometric analysis, confocal images at frequencies of 8, 16, and 22.6 kHz were mapped (Figure 2(a)). For ribbon synapses, spots of CtBP2 staining under each IHC were counted, and the counts of 10 to 12 continuous IHCs at a frequency were averaged for each sample (Figure 2(b)). For MOC efferent synapses [36, 37], the area of SYP staining of each Z-stack maximum intensity projection image was measured and calculated by using ImageJ software (National Institutes of Health, USA, version 1.8.0), which was expressed as the density of MOC efferent synapses of 5 to 6 continuous columns of three rows of outer hair cells (OHCs) for each sample (Figure 2(c)).

### 2.7. Data Processing and Statistical Analysis

Data processing and statistical analyses were performed using GraphPad Prism (GraphPad Software Inc., USA, version 8.0). Continuous variables are presented as the mean (standard deviation, SD) in tables. Cumulative distributions were tested by using the Kolmogorov-Smirnov test. Two-way ANOVA with Bonferroni post hoc tests was used to compare the difference between multiple groups. *P* value < 0.05 was considered statistically significant. In the figures, the error bar represents standard error of the mean (SEM), NS represents *P* > 0.05, ∗ represents *P* < 0.05, and ∗∗ represents *P* < 0.01.

## 3. Results

### 3.1. Dose-Response Relations for Repeated Noise-Induced ABR Threshold Shifts

**Figure 3:**
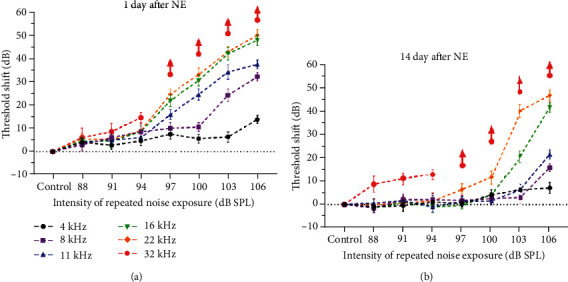
Dose-response relations between repeated noise exposure intensities with ABR threshold shifts at 1 day (a) and 14 days (b) after NE. The error bar represents the SEM for 8-12 mice in each group. Red arrows represent thresholds at 32 kHz frequency greater than 90 dB after the noise exposure.

ABR threshold shifts at 1 day after NE (Figure 3(a)) and 14 days after NE (Figure 3(b)) for groups of repeated noise exposure at various intensities were measured over frequencies from 4 kHz to 32 kHz. No significant threshold shifts were observed in groups of low-intensity noise exposures at 88, 91, and 94 dB SPL at any frequency except for 32 kHz (Table 1). Because the frequency of 32 kHz in C57 mice was extremely vulnerable to hearing loss related to a genetic defect of cadherin in the stereocilia [38, 39], this frequency was excluded from the following analyses in this study. For moderate- to high-intensity repeated noise exposures, threshold shifts at 1 day and 14 days after NE both showed a more striking increase at higher frequencies with intensity, while no significant PTSs showed at the frequency of 4 kHz even under the strongest noise exposure (106 dB SPL) at 14 days after NE. Moderate-intensity noise exposures (97 and 100 dB SPL) induced significant threshold shifts over frequencies from 8 kHz to 22 kHz at 1 day after NE, but no significant PTSs of which except for the 22 kHz in the group of intensity at 100 dB SPL (Table 1).

### 3.2. Not Significant Auditory Effects Induced by Low-Intensity Repeated Noise Exposure

**Figure 4:**
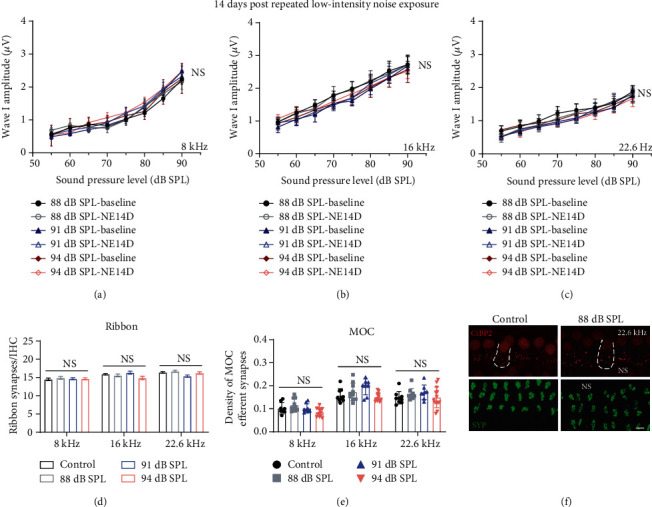
No significant permanent effects on (a–c) ABR wave I amplitudes, (d) ribbon synapse counts, and (e) MOC efferent synaptic measurement post low-intensity (88, 91, and 94 dB SPL) repeated noise exposure. (f) Representative IF images of morphometric analysis at frequency of 22.6 kHz (the scale bar indicates 10 *μ*m). Two-way ANOVA with Bonferroni post hoc tests were used to compare the difference between groups. The error bar represents the SEM for 8-12 mice in each group. NE14D: 14 days after NE; NS: no significance. ^∗^*P* < 0.05; ^∗∗^*P* < 0.01.

For groups of low-intensity noise exposures at 88, 91, and 94 dB SPL without significant threshold shifts of ABR, we further analysed DPOAE threshold shifts (representative group of 88 dB SPL, Supplementary Figure 1), ABR wave I amplitudes (Figures 4(a)–4(c)), ribbon synaptic counts (Figures 4(d) and 4(f)), and the density of MOC efferent synapses (Figures 4(e) and 4(f)) at 14 days after repeated noise exposures, and no significant changes of which were observed at neither 8 kHz, 16 kHz, nor 22.6 kHz frequencies.

### 3.3. Moderate-Intensity Repeated Noise Exposure Impaired Cochlear Synaptic Morphology ahead of Function

**Figure 5:**
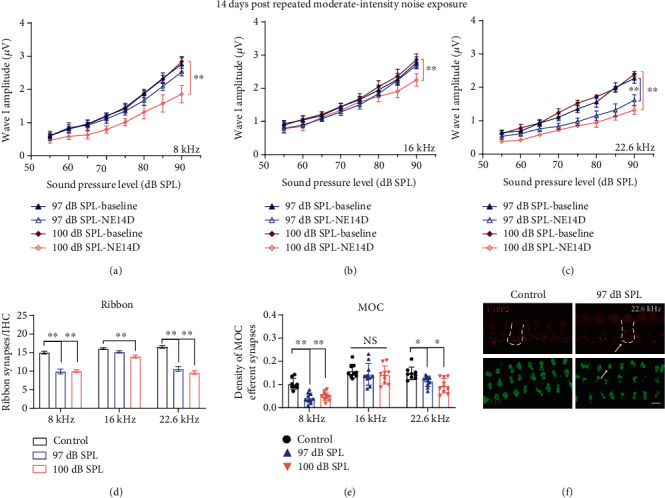
Moderate-intensity (97 and 100 dB SPL) repeated noise-induced permanent effects on (a–c) ABR wave I amplitudes, (d) ribbon synapse counts, and (e) MOC efferent synaptic quantification. (f) Representative IF images of morphometric analysis for group 97 dB SPL at a frequency of 22.6 kHz; white arrows indicate significant morphometric changes (the scale bar indicates 10 *μ*m). Two-way ANOVA with Bonferroni post hoc tests were used to compare the difference between groups. The error bar represents the SEM for 8-12 mice in each group. NE14D: 14 days after NE; NS: no significance. ^∗^*P* < 0.05; ^∗∗^*P* < 0.01.

For moderate-intensity noise exposures, the group of 97 dB SPL showed only TTSs at frequencies of 8, 16, and 22.6 kHz, while the group of 100 dB SPL showed more serious threshold shifts than that in the 97 dB SPL group and even a PTS (ABR and DPOAE threshold shifts) at the frequency of 22.6 kHz (Supplementary Figure 1). The frequency of 22.6 kHz was most vulnerable to repeated acoustic trauma on ABR wave I amplitudes, ribbon synapses, and MOC efferent synapses in groups of moderate-intensity noise exposures (Figures 5(c)–5(f)). Despite the considerable degree of threshold shifts at 1 day after NE (averaged 21.82 dB for group 97 dB SPL and 30.5 dB for group 100 dB SPL), 16 kHz was the most robust frequency against the synaptic degeneration from repeated noise exposures at 97 dB SPL; however, it showed a mild but significant decrease of wave I amplitudes and ribbon synaptic counts in the 100 dB SPL group (Figures 5(b) and 5(d)). Notably, changes at a frequency of 8 kHz indicated that the lower moderate-intensity (97 dB SPL) repeated noise exposures induced the cochlear ribbon and MOC efferent synaptic degeneration (Figures 5(d) and 5(e)) before ABR wave I amplitudes decreased (Figure 5(a)).

### 3.4. High-Intensity Repeated Noise Exposure Impaired Outer Hair Cells despite Cochlear Synaptic Degeneration

**Figure 6:**
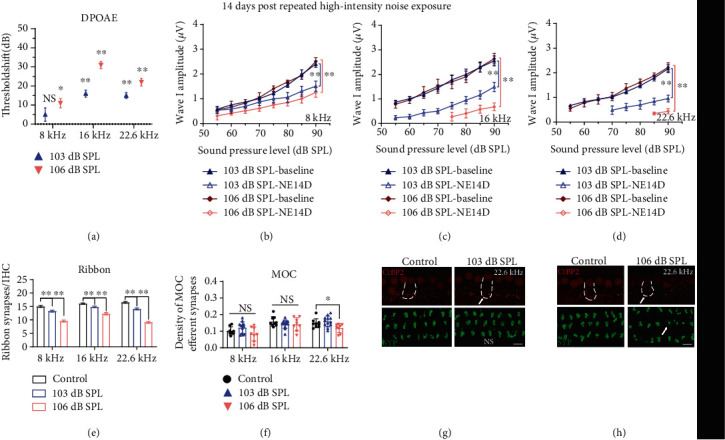
High-intensity (103 and 106 dB SPL) repeated noise-induced permanent effects on (a) DPOAE, (b–d) ABR wave I amplitudes, (e) ribbon synapse counts, and (f) MOC efferent synaptic quantification. Representative IF images of morphometric analysis for groups (g) 103 dB SPL and (h) 106 dB SPL at the frequency of 22.6 kHz; white arrows indicate significant morphometric changes (the scale bar indicates 10 *μ*m). Two-way ANOVA with Bonferroni post hoc tests were used to compare the difference between groups. The error bar represents the SEM for 8-12 mice in each group. NE14D: 14 days after NE; NS: no significance. ^∗^*P* < 0.05; ^∗∗^*P* < 0.01.

In consideration of PTSs induced by repeated high-intensity noise exposures, we performed DPOAE tests and HC counting at 14 days post exposures. No significant loss of HCs was found, even for the group of highest intensity (Supplementary Figure 2), while significant DPOAE threshold shifts were essentially consistent with PTSs at frequencies of 8, 16, and 22.6 kHz for each group (Table 1 and Figure 6(a)). In accordance with expectations, ABR wave I amplitudes and ribbon synaptic counts permanently reduced in high-intensity noise-exposed groups (Figures 6(b)–6(e)). However, to our surprise, the decrement of MOC efferent synapses was not significant after repeated noise exposures at high intensities except for that at the frequency of 22.6 kHz in the 106 dB SPL group (Figures 6(f)–6(h)).

## 4. Discussion

In this study, we explored and summarized the dose-dependent pattern of cochlear functional and morphological degeneration induced by repeated noise exposure at various intensities from 88 to 106 dB SPL in a 3 dB step increment.

Despite numerous studies that have demonstrated noise-induced cochlear synaptic degeneration in various animal models [18, 19, 25, 39–42], most of them used a single, short-duration noise exposure procedure extensively used in CBA/CaJ mice, Sprague-Dawley rats, guinea pigs, etc. The C57BL/6 strain mouse was not commonly used in previous NIHL studies, because it showed more severe ABR and DPOAE threshold shifts at high frequencies compared with CBA mice [39, 43], which was attributed to the genetic defect in the stereocilia of carrying Cdh23^ahl^ alleles [38]. However, in recent years, since many laboratories have moved their mutant genes of interest to the C57BL/6 background [44], this strain was widely used for genetic studies, including many NIHL studies [45–47]. Moreover, previous strain comparisons revealed that C57 mice were more susceptible than CBAs in the older age group only [43], while a recent study indicated that the susceptibility of noise-induced cochlear ribbon synaptopathy in CBA mice was different from C57 mice [31]. Thus, we chose to use young C57 mice aged four weeks and followed up to the age of 7 weeks. In this study, we provided the characterization of repeated noise-induced injury to cochlear function, synaptic morphology, and their dose-response relationships in C57BL/6J mice.

### 4.1. Smaller TTS Did Not Show Evidence of Cochlear or Synapse Pathology with Repeated Exposure

Given the compelling evidence that even moderate noise exposure can result in cochlear synaptic degeneration, numerous studies asked whether prolonged overexposure to various noise levels that had been considered “harmless” would add significant risk to NIHL [48, 49]. One challenge in understanding the cochlear consequence of noise is to overview damage patterns of the wide range of possible stimulus parameters. Referring to human daily exposures, we wondered if cochlear synaptic degeneration results from repeated exposures at lower SPLs.

Overall, in this study, one-week long repeated noise exposure at relatively low intensities seemed to be benign for young C57BL/6J mice during a moderate period (two weeks in our results). The “low” intensities (up to 94 dB SPL) of broadband noise used in this study are remarkably higher than that of environmental sounds, which did not induce even temporary impairments on cochlear function and synaptic morphology. These results could be supported by some previous studies. Morgan et al. [27] exposed Sprague-Dawley rats to 8-16 kHz octave-band noise at 97 dB SPL for 2 hours, which repeated for 4 consecutive days. They demonstrated that daily repeated exposures result in diminished TTS and recovered thresholds; moreover, no permanent reduction in suprathreshold ABR responses was observed. Mannström et al. exposed female Sprague-Dawley rats to 2-20 kHz broadband noise for 1.5 hours at various intensities, which was repeated every six weeks. They found that rats exposed to the repeated noise exposure at 101 and 104 dB SPL did not have any permanent impairment in thresholds or ABR wave I amplitudes in comparison with unexposed control rats [50]. Despite the species differences in the noise dose required to generate cochlear injuries [51, 52], our results suggested that in C57 mice [27], there is also a permissible dose of noise exposure that does not directly cause the significant TTS as well as the cochlear synaptic degeneration. However, the long-term effects of accumulated noise-induced trauma in cochlea synapses should be further considered in future studies.

### 4.2. Damage Pattern of Repeated Noise Exposures

Noise-induced cochlear damage may take various patterns underlying different degrees and mechanisms for reversible or permanent impairments. To date, the sustained cochlear damage across all mammalian species studies seems to progress similarly with the noise dose increase, which first occurs in IHC ribbon synapses, then the stereocilia, later the loss of HCs and ANs [52, 53]. Different from the single octave band of noise extensively used in many previous studies [17, 18, 22], we used the repeated broadband noise at intensities from 97 to 106 dB SPL without producing significant loss of HCs, in order to focus on the cochlear synaptic degeneration and dysfunction of OHC stereocilia (reflected on DPOAE) and their vulnerability at various frequencies. Notably, we took not only ribbon synapses but also MOC efferent synapses into consideration of the cochlear synaptic degeneration.

As expected, repeated noise-induced hearing threshold shifts in C57 mice were more severe at higher frequencies, which should be attributed to the dysfunction or damage of OHC stereocilia. In this study, we further found that the vulnerability to repeated noise-induced cochlear synaptic degeneration was more remarkable at the higher frequency of 22.6 kHz and the lower frequency of 8 kHz, while the middle frequency of 16 kHz was most robust against synaptic degeneration. For all frequencies, TTSs first occurred with the increased doses of repeated noise exposure, which were not always accompanied by synaptic degeneration. Moreover, the change of ribbon synaptic counts appeared to be consistent with MOC efferent synapses under low- and moderate-intensity noise exposure. However, PTSs accompanied by damage on DPOAEs were more likely to result in loss of ribbon synapses rather than MOC efferent synapses (Table 2). To our knowledge, this present study showed for the first time that repeated noise exposure leading to cochlear synaptic degeneration could also cause reduction of MOC efferent synapses, which depended on the vulnerability of frequency and function of OHCs.

As previous studies indicated, the relationship between threshold shifts at 1 day after NE criterion change and ABR amplitudes or synaptic counts at each frequency was quite complicated [22, 51, 54, 55]. Among Sprague-Dawley rats, only the 8-16 kHz bandpass noise exposures producing TTSs at 1 day after NE greater than 30 dB could reduce ABR wave I amplitudes, while the degree of ABR wave I reduction was not related to the degree of threshold shifts [19]. However, Maison et al. reported significant cochlear ribbon synaptopathy in CBA mice exposed to lower intensity but more prolonged octave noise that only produced a 15 dB TTS [29]. Here, we found that the degree of threshold shifts at 1 day after NE producing cochlear synaptic degeneration was particularly related to the frequency. Our results suggested that approximately 10 dB at 8 kHz, 30 dB at 16 kHz, and 20 dB at 22.6 kHz of threshold shifts at 1 day post exposure are able to result in permanent loss of ribbon synapses. Moreover, the degree of threshold shifts at 1 day after NE progress following PTSs was about 30 dB at 8 kHz and 22.6 kHz, which was higher than 40 dB at a frequency of 16 kHz (Table 1). The findings suggested that repeated or prolonged noise exposure might cause greater cochlear synaptic degeneration at lower and higher frequencies, in the order of ARHL patterns of IHC synapse loss observed in human temporal bones [56, 57]. Fernandez et al. previously demonstrated that CBA/CaJ mice exposed to 8-16 kHz noise at 91 dB SPL for 2 hours or 8 hours produced no loss of synapses at 16 kHz and below, while the synaptic loss increased with frequency for 8-hour exposure compared with the 2-hour exposure [22]. Repeated exposures to the single noise that induced only TTSs resulted in cumulative cochlear ribbon synaptopathy at frequencies of 16 kHz and above as well [58]. These results suggested that repeated noise overstimulation probably accelerates the cochlear synaptic degeneration in the animal model of ARHL [59].

### 4.3. Potential Effects on MOC Efferent Synapses of Repeated Noise Exposure

It is widely accepted that the feedback from the MOC efferent system can protect cochlear ribbon synaptopathy from both acute and chronic noise exposures [29, 60, 61]. Maison et al. removed all efferent feedback to the inner ear by cutting the efferent bundles, whereas the sectioning of the efferent fibers greatly exacerbated the ribbon synaptopathy in both basal and apical regions of the cochlea in CBA/CaJ mice under one-week exposure at 84 dB SPL [29]. A recent study used mice with a gain-of-function point mutation in the *α*9 subunit of the nicotinic acetylcholine receptor, which strengthened cochlear suppression of the MOC efferent system protecting the loss of ribbon synapses from acoustic injury [62]. The aging-induced MOC system decline has been demonstrated in numerous previous studies. The density of MOC efferent terminals decreased with age prior to OHC degeneration, as measured by contralateral suppression (CS) of DPOAEs in humans and CBA mice [63, 64].

However, it remains unclear whether noise exposure results in damage to MOC efferent nerves. Only a few studies have focused on damage to efferent nerve endings following noise exposure. Although the previous work failed to observe any effects on CS of acute recreational noise exposure in normal hearing threshold adults [65], Boero et al. first demonstrated that acute 1-16 kHz noise exposure at 100 dB SPL for 1 hour can produce degeneration of MOC terminals contacting the OHCs [60]. Consistently in this study, we first demonstrated that repeated noise exposure in C57 mice also results in MOC efferent synaptic degeneration. Our results indicated that the MOC efferent synapses showed strong resistance to noise damage, as well as partial protection from ribbon synaptopathy at middle frequencies of 16 kHz (Table 2), in accordance with the distribution of MOC terminals as previous observations [63, 66]. Further studies to reveal repeated noise-induced functional changes of MOC efferent nerves need to be performed in the future.

Notably, we found that various repeated noise-induced effects on patterns of TTSs, PTSs, ABR wave I amplitudes, ribbon, and MOC efferent synaptic degeneration were quite complex. For instance, although both ribbon and MOC efferent synapses decreased at frequency of 8 kHz, ABR wave I amplitudes reduced in group 100 dB SPL rather than 97 dB SPL (Figure 4). We proposed that the different damage patterns may depend on balance of the degree of injury of various inner ear elements, especially the MOC efferent feedback. Besides, repeated high-intensity noise exposures enabled production of PTSs unexpectedly resulting in slighter MOC efferent synaptic degeneration than that under moderate-intensity noise. These results suggested that noise-induced PTS may alter synaptopathic outcomes. Fernandez et al. recently assessed the dose-response effects on ribbon synaptopathy and HC damage of acute 8-16 kHz octave-band noise exposure in CBA/CaJ mice. They also observed that higher-level noise exposure producing mixed sensory and neural loss resulted in smaller synapse losses, despite greater declines in suprathreshold ABR amplitudes [53]. Underlying mechanisms might involve HC injury attenuating the direct stimulus on synapses, which protected them from synaptic excitotoxicity [17].

## 5. Conclusions

In summary, we demonstrated the dose-dependent characterization of the repeated noise-induced injury to cochlear function, synaptic morphology, and their complex dose-response relationships in C57BL/6J mice. We proposed that the noise-induced various cochlear damage patterns attribute to the balance of degrees of injury on HCs, ribbon and MOC efferent synapses, etc. Notably, this study provided a sight into the hypothesis that the interruption in synaptic communication between MOC efferent terminals and OHCs, together with loss of ribbon synapses, contributes to prolonged noise-induced cochlear synaptic degeneration.

## Figures and Tables

**Table 1:** ABR threshold shifts at 1 day and 14 days after repeated noise exposure at various intensities.

Intensity (dB SPL)	ABR threshold shifts (dB HL)											
	4 kHz		8 kHz		11.3 kHz		16 kHz		22.6 kHz		32 kHz	
	Mean (SD)	*P* value	Mean (SD)	*P* value	Mean (SD)	*P* value	Mean (SD)	*P* value	Mean (SD)	*P* value	Mean (SD)	*P* value
At 1 day after NE												
88 (*n* = 10)	4.00 (5.68)	0.3699	3.00 (8.56)	>0.9999	4.00 (4.59)	0.7544	4.50 (4.97)	0.131	5.50 (6.85)	0.2018	6.00 (13.08)	—
91 (*n* = 11)	2.73 (6.07)	>0.9999	6.00 (8.43)	0.357	5.00 (5.00)	0.5525	4.50 (9.26)	>0.9999	5.00 (8.50)	0.6249	8.50 (11.32)	—
94 (*n* = 11)	4.55 (6.88)	0.474	8.50 (10.55)	0.2195	5.91 (6.25)	0.0883	8.50 (7.84)	0.0525	9.00 (9.37)	0.0877	14.55 (7.23)	—
97 (*n* = 12)	7.50 (7.54)	0.0664	**10.00 (8.79)**	**0.0468**	**15.83 (7.64)**	**0.0381**	**21.82 (8.15)**	**<0.0001**	**24.58 (7.82)**	**<0.0001**	—	—
100 (*n* = 10)	5.50 (5.99)	0.1221	**10.50 (5.99)**	**0.0025**	**24.50 (7.62)**	**0.0006**	**30.50 (7.25)**	**<0.0001**	**33.00 (9.19)**	**<0.0001**	—	—
103 (*n* = 12)	6.25 (7.72)	0.1999	**24.17 (9.49)**	**0.0002**	**34.17 (11.04)**	**0.0013**	**42.08 (9.16)**	**<0.0001**	**42.92 (7.82)**	**<0.0001**	—	—
106 (*n* = 8)	**13.75 (5.18)**	**0.0007**	**32.14 (4.88)**	**<0.0001**	**37.50 (5.35)**	**0.0006**	**47.86 (6.36)**	**<0.0001**	**50.00 (7.50)**	**<0.0001**	—	—
At 14 days after NE												
88 (*n* = 10)	-1.00 (5.16)	>0.9999	-1.50 (6.26)	>0.9999	0.50 (5.50)	>0.9999	-1.50 (5.80)	>0.9999	0.00 (8.16)	>0.9999	8.50 (10.55)	—
91 (*n* = 11)	-0.50 (7.62)	>0.9999	2.00 (7.53)	>0.9999	1.50 (6.26)	>0.9999	1.00 (3.94)	>0.9999	0.45 (8.79)	>0.9999	10.91 (8.61)	—
94 (*n* = 11)	1.00 (8.43)	>0.9999	2.00 (8.56)	>0.9999	-1.00 (7.38)	>0.9999	-1.00 (6.58)	>0.9999	1.50 (10.55)	>0.9999	12.73 (7.54)	—
97 (*n* = 12)	0.42 (5.42)	>0.9999	1.67 (6.85)	>0.9999	1.25 (6.78)	>0.9999	-0.42 (4.98)	>0.9999	6.25 (8.56)	0.321	—	—
100 (*n* = 10)	4.00 (6.99)	0.7272	2.50 (6.35)	>0.9999	1.50 (5.30)	>0.9999	4.00 (6.58)	0.6079	**11.5 (9.44)**	**0.0273**	—	—
103 (*n* = 12)	6.25 (8.56)	0.3317	2.92 (8.65)	>0.9999	6.25 (8.01)	0.2999	**20.42 (8.38)**	**0.0003**	**40.00 (9.29)**	**<0.0001**	—	—
106 (*n* = 8)	7.14 (6.36)	0.1267	**15.71 (4.50)**	**0.0004**	**21.43 (5.56)**	**0.0002**	**41.43 (5.56)**	**<0.0001**	**46.67 (6.06)**	**<0.0001**	—	—

Two-way ANOVA with Bonferroni post hoc tests were used to compare the difference between ABR thresholds at 1 day or 14 days after NE with the baseline in each group of intensity. Bold type: *P* < 0.05.

**Table 2:** Cochlear function and synaptic morphology changes related to repeated noise exposure at various intensities.

Frequency	Intensity (dB SPL)		Function			Synaptic morphology	
			Threshold shifts at 1 day after NE	Threshold shifts at 14 days after NE	Decreased ABR wave I amplitude	Ribbon synaptopathy	MOC efferent synaptic degeneration
8 kHz	Low	88, 91, 94	(-)	(-)	(-)	(-)	(-)
	Moderate	97	(+)	(-)	(-)	(+)	(+)
		100	(+)	(-)	(+)	(+)	(+)
	High	103	(+)	(-)	(+)	(+)	(-)
		106	(+)	(+)	(+)	(+)	(-)

16 kHz	Low	88, 91, 94	(-)	(-)	(-)	(-)	(-)
	Moderate	97	(+)	(-)	(-)	(-)	(-)
		100	(+)	(-)	(+)	(+)	(-)
	High	103	(+)	(+)	(+)	(+)	(-)
		106	(+)	(+)	(+)	(+)	(-)

22 kHz	Low	88, 91, 94	(-)	(-)	(-)	(-)	(-)
	Moderate	97	(+)	(-)	(+)	(+)	(+)
		100	(+)	(+)	(+)	(+)	(+)
	High	103	(+)	(+)	(+)	(+)	(-)
		106	(+)	(+)	(+)	(+)	(+)

(+) indicates significant change; (-) indicates nonsignificant change.

## Data Availability

The analysed data used to support the findings of this study are included within the article; further inquiries are available from the corresponding authors upon request.

## Supplementary Materials

Supplementary Materials Supplementary Figure 1: representative low-intensity (88 dB SPL) and moderate-intensity (97 and 100 dB SPL) repeated noise-induced DPOAE threshold shifts at 14 days after NE. Two-way ANOVA with Bonferroni post hoc tests were used to compare the difference compared with baseline. The error bar represents the SEM for 8-12 mice in each group. NS: no significance; ^∗^*P* < 0.05. Supplementary Figure 2: whole-mount cochlear immunofluorescence at 14 days after 106 dB SPL repeated noise exposure. No significant HC loss was observed at frequencies of 8, 16, or 22.6 kHz. The scale bar indicates 20 *μ*m.
