# Supplementary material for: Dose-Dependent Pattern of Cochlear Synaptic Degeneration in C57BL/6J Mice Induced by Repeated Noise Exposure
Source: Neural Plast. 2021 Jun 9;2021:9919977. doi: 10.1155/2021/9919977 (PMC8211526; doi:10.1155/2021/9919977)
Supplement: Supplementary Materials — Supplementary Figure 1: representative low-intensity (88 dB SPL) and moderate-intensity (97 and 100 dB SPL) repeated noise-induced DPOAE threshold shifts at 14 days after NE. Two-way ANOVA with Bonferroni post hoc tests were used to compare the difference compared with baseline. The error bar represents the SEM for 8-12 mice in each group. NS: no significance; ∗P < 0.05. Supplementary Figure 2: whole-mount cochlear immunofluorescence at 14 days after 106 dB SPL repeated noise exposure. No significant HC loss was observed at frequencies of 8, 16, or 22.6 kHz. The scale bar indicates 20 μm. [file 9919977.f1.docx]

## Supplementary Materials

Supplementary figure 1: Representative low-intensity (88 dB SPL) and moderate-intensity (97- and 100 dB SPL) repeated noise-induced DPOAE threshold shifts at 14 days after NE. Two-way ANOVA with Bonferroni post-hoc tests were used to compare the difference compared with baseline. The error bar represents the SEM for 8 - 12 mice in each group. NS: no significance, **P* < 0.05.

Supplementary figure 2: Whole-mount cochlear immunofluorescence at 14 days after 106 dB SPL repeated noise exposure. No significant HCs loss were observed at frequencies of 8, 16 or 22.6 kHz. The scale bar indicates 20μm.
